# Intraabdominal pressure in critical burn patients

**DOI:** 10.1186/cc14466

**Published:** 2015-03-16

**Authors:** PM Millan

**Affiliations:** 1 Hospital Universitario La Paz, Madrid, Spain

## Introduction

The aim was to study the evolution and incidence of intraabdominal hypertension in critical burn patients using a slightly restrictive fluid therapy protocol based on monitoring transpulmonary thermodilution and lactic acid.

## Methods

A prospective study of 132 consecutive patients admitted to the Critical Burn Unit between October 2008 and October 2011. In all of them resuscitation was performed by objectives: blood pressure (>65 mmHg), hourly diuresis (0.5 to 1 cm^3^/kg), lactic acid clearance and thermodilution transpulmonary parameters (CI >2.5 l/minute/m^2^, ITBI: 600 ml/m^2^). We performed measurements of IAP with a bladder catheter every 8 hours in the first 72 hours.

## Results

**Figure 1:**
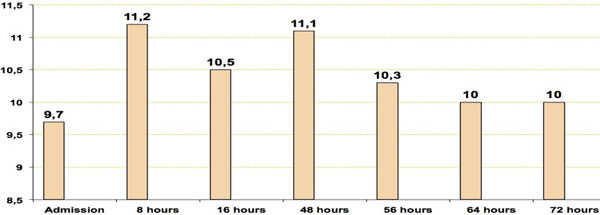
**Intraabdominal pressure**.

**Figure 2:**
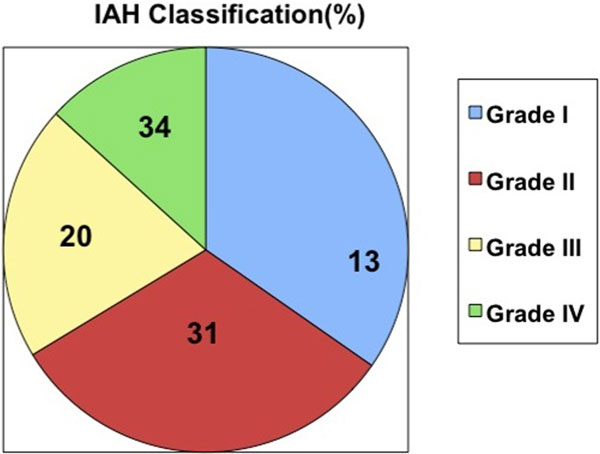
**IAH classification**.

Ninety-eight men and 34 women were studied. Mean age 48 ± 18 years and a TBSA of 35 ± 22%. The fluid provided by %TBSA in the first 8 hours was less than predicted by Parkland (4.05 ml/kg), although the total contribution in the first 24 hours was similar. The evolution of the intra-abdominal pressure was: admission 9.7 mmHg, 8 hours 11, 16 hours 10.5, 24 hours 12.1, 32 hours 12.0, 40 hours 12.0, 48 hours 11.1, 56 hours 10.3, 64 hours 10.0 and 72 hours 10.0. A total of 44 patients (33.3%) had a determination higher than 12 mmHg, distributed: 15 patients between 12 and 15 mmHg (IAHT I grade), 14 between 16 and 20 mmHg (II), nine between 21 and 25 mmHg (III) and six >25 mmHg (IV). See Figures 1 and 2.

## Conclusion

IAH incidence when a slightly restrictive fluid protocol used is less than expected.

